# Angiotensin-Converting Enzyme (ACE) Inhibitor-Associated Hypersensitivity Vasculitis With Small Bowel Edema: A Case Report

**DOI:** 10.7759/cureus.103653

**Published:** 2026-02-15

**Authors:** Lamyae Debbagh, Marc Zalcman

**Affiliations:** 1 Radiology, Université Libre de Bruxelles, Brussels, BEL; 2 Radiology, Université Libre de Bruxelles/Hôpital Erasme, Brussels, BEL

**Keywords:** ace inhibitor, hypersensitivity vasculitis, intestinal angioedema, non-ischemic bowel edema, small bowel edema

## Abstract

Angiotensin-converting enzyme (ACE) inhibitor-associated hypersensitivity vasculitis with gastrointestinal involvement is an uncommon but important drug-related cause of acute abdominal pain and may mimic an acute surgical abdomen. We report the case of an 84-year-old man admitted for redo aortic valve replacement following infective endocarditis, who developed acute abdominal pain on postoperative day 14. Physical examination revealed diffuse abdominal tenderness and cutaneous purpura. Laboratory testing showed elevated inflammatory markers with peripheral eosinophilia. Contrast-enhanced abdominal CT demonstrated concentric small bowel wall thickening with submucosal edema and ascites, without evidence of mesenteric ischemia. Skin biopsy confirmed leukocytoclastic vasculitis. Lisinopril was initiated at the time of admission and continued throughout the perioperative period; symptoms occurred approximately three weeks after treatment initiation, with no prior history of similar episodes. Discontinuation of lisinopril resulted in rapid clinical improvement and complete resolution of abdominal symptoms. This case highlights that ACE inhibitor-associated hypersensitivity vasculitis may present with small bowel edema and imaging findings overlapping with intestinal angioedema. Careful medication review and recognition of associated systemic features such as purpura and eosinophilia are essential to avoid unnecessary invasive procedures.

## Introduction

Angiotensin-converting enzyme inhibitors (ACEIs) are widely prescribed for the treatment of hypertension, heart failure, and cardiovascular protection. Angioedema is a well-recognized adverse effect, usually involving the lips, tongue, or airway, and occurs in approximately 0.1-0.7% of patients receiving ACEIs [1,2]. Visceral involvement is a much rarer manifestation, first described in the 1980s, and remains underdiagnosed due to its nonspecific symptoms and delayed onset after drug initiation [3,4]. Patients typically present with acute abdominal pain, nausea, vomiting, or diarrhea, often mimicking mesenteric ischemia, inflammatory bowel disease, or intestinal obstruction [5-7]. Imaging, particularly contrast-enhanced CT, plays a crucial role in diagnosis, showing segmental bowel wall thickening, submucosal edema, and ascites [8,9]. Although ACEI-related bowel edema is classically attributed to bradykinin-mediated mechanisms, ACEIs may also rarely be associated with immune-mediated hypersensitivity reactions, including leukocytoclastic vasculitis, which may involve the gastrointestinal tract and produce overlapping imaging findings. Rapid resolution of symptoms following discontinuation of the ACEI strongly supports the diagnosis [6,10]. Awareness of this entity is essential, as misdiagnosis may lead to unnecessary surgical interventions. We report a case of lisinopril-associated leukocytoclastic vasculitis with gastrointestinal involvement presenting with small bowel edema, highlighting diagnostic challenges and the importance of careful medication review in elderly postoperative patients with acute abdominal pain.

## Case presentation

An 84-year-old man with a history of aortic valve replacement in 2011 and a permanent pacemaker was admitted for redo aortic valve surgery following infective endocarditis. His immediate postoperative course was uneventful until day 14, when he developed severe intermittent abdominal pain rated 7/10 on the visual analogue scale. The pain was associated with nausea and vomiting but occurred in the absence of fever, diarrhea, or constipation. On clinical examination, the patient presented with diffuse abdominal tenderness and guarding. In addition, a progressive purpuric rash appeared on the wrists and arms and extended to the upper trunk during hospitalization. Vital signs remained stable throughout the episode. Laboratory investigations demonstrated elevated inflammatory and hematological parameters, as summarized in Table 1.

**Table 1:** Laboratory investigations. CRP: C-reactive protein; LDH: lactate dehydrogenase; WBC: white blood cell count Units: mg/L (milligrams per liter); /µL (cells per microliter); U/L (units per liter); ×10⁹/L (10⁹ cells per liter). Reference ranges derived from standard hospital laboratory values and commonly cited reference intervals [11,12].

Parameter	Result	Reference range
C-reactive protein (CRP)	88 mg/L	<5 mg/L
White blood cells	12,000 /µL	4,000-10,000 /µL
LDH (current)	389 U/L	135-225 U/L
LDH (previous)	447 U/L	135-225 U/L
Eosinophils	Elevated	<0.5 × 10⁹/L

**Figure 1:**
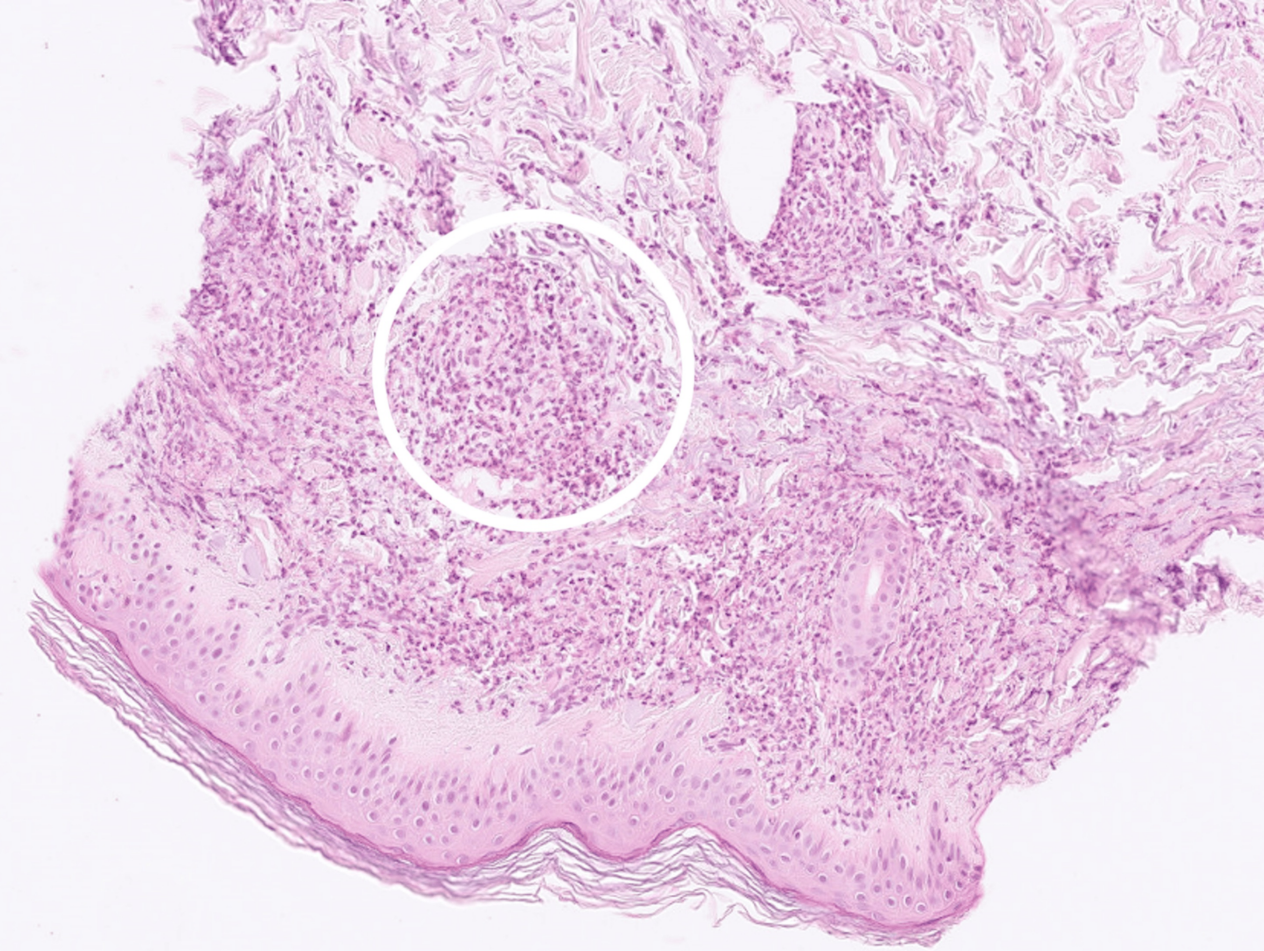
Histopathology of skin biopsy. Hematoxylin and eosin stain, skin biopsy, ×10 magnification. The specimen shows features of leukocytoclastic vasculitis characterized by dense neutrophilic infiltration of dermal vessels with perivascular inflammation; one representative affected area is highlighted by a white circle.

**Figure 2:**
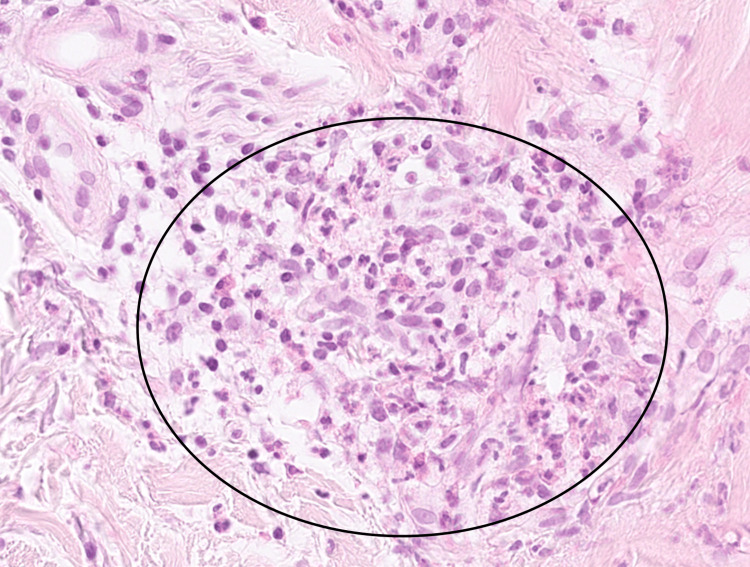
Histopathology of skin biopsy at high magnification. Hematoxylin and eosin stain, skin biopsy, ×40 magnification. The image demonstrates leukocytoclastic vasculitis with prominent neutrophilic infiltration and perivascular inflammatory changes (black circle).

Histopathological analysis of the skin biopsy confirmed the presence of neutrophilic leukocytoclastic vasculitis involving dermal vessels (Figures 1-2).

**Figure 3:**
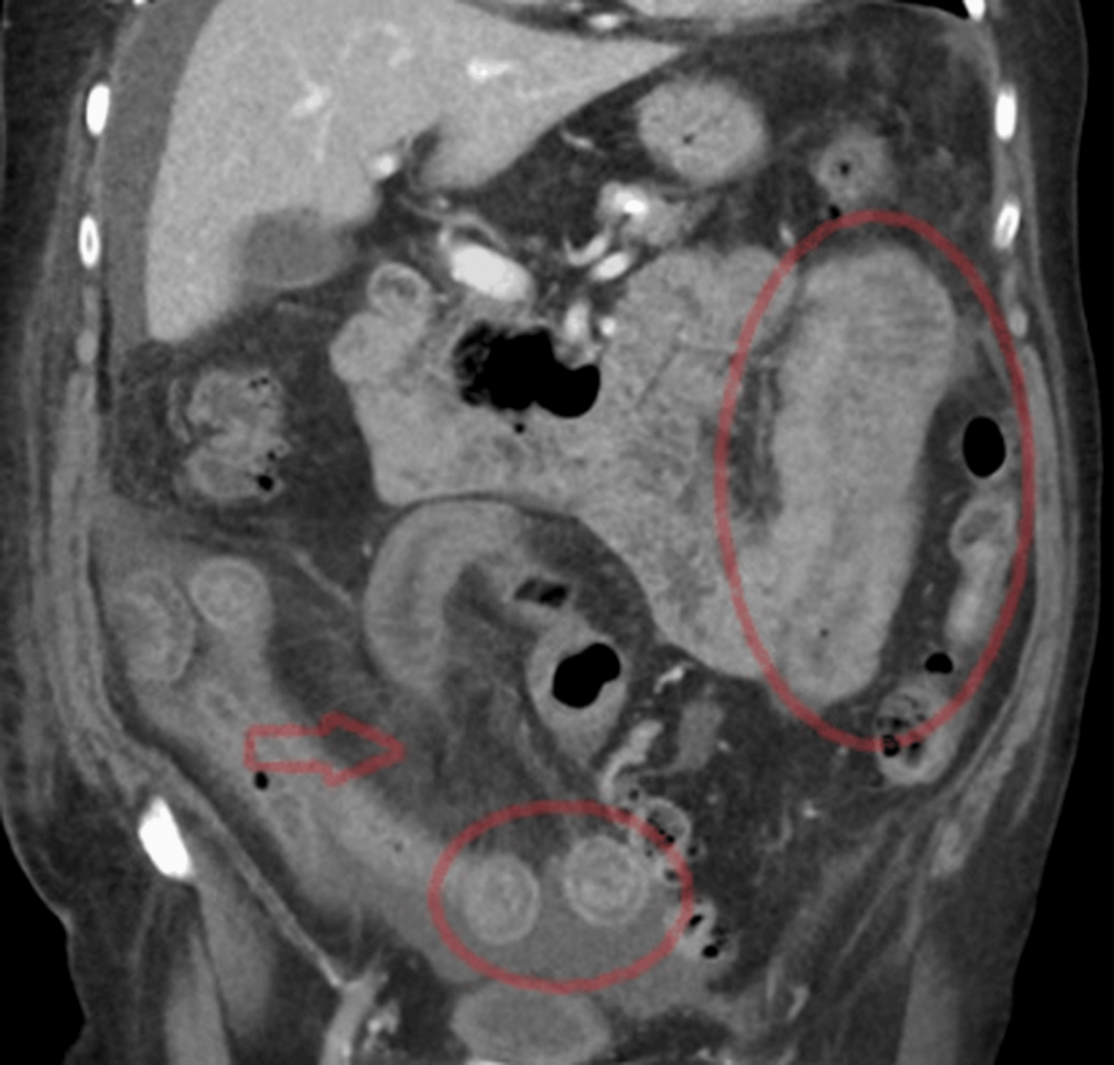
Coronal contrast-enhanced CT images showing concentric small bowel wall thickening with submucosal edema and stratified enhancement of the small bowel loops (red ellipses) with mesenteric comb sign (red arrow).

**Figure 4:**
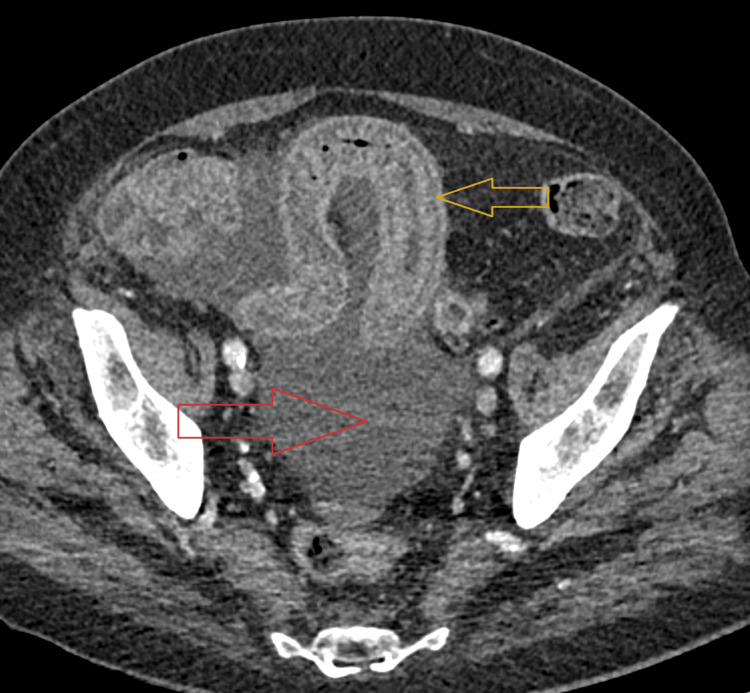
Axial contrast-enhanced CT image of the pelvis showing marked bowel wall edema (yellow arrow) with ascites (red arrow).

Urgent contrast-enhanced abdominal CT demonstrated a long segment of concentric small bowel wall thickening associated with submucosal edema, stratified mural enhancement, and the mesenteric comb sign. While the mesenteric comb sign and stratified mural enhancement are not specific and can be observed in inflammatory bowel disease or enteritis, the concurrent systemic findings in our patient (purpura, eosinophilia, and skin biopsy-confirmed leukocytoclastic vasculitis) supported the diagnosis of an immune-mediated hypersensitivity vasculitis with gastrointestinal involvement, which may produce CT findings similar to intestinal angioedema. Moderate ascites was also present, while no signs of mesenteric ischemia or mechanical obstruction were identified (Figures 3-4).

Based on the clinical and radiological findings, the internal medicine team suspected lisinopril-induced hypersensitivity vasculitis with intestinal involvement. Discontinuation of lisinopril resulted in the rapid resolution of abdominal symptoms and complete clinical recovery.

## Discussion

ACE inhibitor-related gastrointestinal involvement is uncommon but clinically important, as it may present with acute abdominal pain and imaging findings suggestive of an acute surgical abdomen. Visceral edema associated with ACE inhibitors is classically attributed to bradykinin accumulation, resulting in increased vascular permeability and submucosal bowel edema [1,2]. Clinically, patients typically present with nonspecific symptoms such as abdominal pain, nausea, vomiting, and, in some cases, diarrhea. Because of this nonspecific presentation, the condition is frequently misdiagnosed as mesenteric ischemia, inflammatory bowel disease, or intestinal lymphoma [3-6].

However, ACE inhibitors may also rarely trigger immune-mediated hypersensitivity reactions, including leukocytoclastic vasculitis, which can involve the gastrointestinal tract and lead to similar clinical and radiological presentations. In our patient, the presence of purpura, peripheral eosinophilia, and biopsy-proven leukocytoclastic vasculitis strongly supported an immune-complex hypersensitivity vasculitis with gastrointestinal involvement rather than isolated bradykinin-mediated intestinal angioedema.

Radiological imaging, particularly contrast-enhanced abdominal CT, plays a key role in the diagnostic workup. Typical findings include segmental concentric bowel wall thickening, prominent submucosal edema with stratified mural enhancement, and variable amounts of ascites, usually without evidence of vascular occlusion or bowel ischemia [7,8]. Nevertheless, these CT features are not specific and may also be observed in inflammatory enteritis or vasculitis, making correlation with clinical and laboratory findings essential [3-6]. Recognition of this entity remains important to avoid unnecessary exploratory surgery, and rapid clinical improvement following ACEI discontinuation strongly supports a drug-related mechanism [4,6,9].

Although a drug rechallenge was not performed for safety reasons, the temporal relationship with lisinopril initiation and the rapid resolution after treatment discontinuation further supported the suspected diagnosis. This case highlights the value of careful medication review and recognition of associated systemic features in elderly postoperative patients presenting with acute abdominal pain, in order to prevent unnecessary invasive investigations and surgical exploration.

## Conclusions

For this patient, although a rechallenge was not performed for safety reasons, the suspected diagnosis of lisinopril-associated hypersensitivity vasculitis with gastrointestinal involvement was supported by the temporal relationship with treatment initiation, purpura with biopsy-proven leukocytoclastic vasculitis, compatible CT findings of small bowel edema, and rapid improvement after drug discontinuation. This case illustrates that in elderly postoperative patients presenting with acute abdominal pain, drug-induced hypersensitivity reactions should be considered before pursuing invasive procedures. It also highlights the value of careful medication review and radiological assessment in preventing unnecessary interventions.

## References

[1] Oudit G, Girgrah N, Allard J (2001). ACE inhibitor-induced angioedema of the intestine: case report, incidence, pathophysiology, diagnosis and management. Can J Gastroenterol.

[2] Baram M, Kommuri A, Sellers SA, Cohn JR (2013). ACE inhibitor-induced angioedema. J Allergy Clin Immunol Pract.

[3] Corda DM, Dexter F, Pasternak JJ, Trentman TL, Nottmeier EW, Brull SJ (2011). Patients' perspective on full disclosure and informed consent regarding postoperative visual loss associated with spinal surgery in the prone position. Mayo Clin Proc.

[4] Kotlyar D, Hirten R, Pasamba M (2010). Angioedema of the bowel with ACE inhibitors: case report and systematic review. Am J Gastroenterol.

[5] Sravanthi MV, Suma Kumaran S, Sharma N, Milekic B (2020). ACE inhibitor induced visceral angioedema: an elusive diagnosis. BMJ Case Rep.

[6] Wilin KL, Czupryn MJ, Mui R, Renno A, Murphy JA (2018). ACE inhibitor-induced angioedema of the small bowel: a case report and review of the literature. J Pharm Pract.

[7] Campbell T, Peckler B, Hackstadt RD, Payor A (2010). ACE inhibitor-induced angioedema of the bowel. Case Rep Med.

[8] Barnett J, Yu KK, Mayilvaganan B (2024). Small bowel angioedema secondary to ACE inhibitor. Am J Gastroenterol.

[9] Suenghataiphorn T, Tribuddharat N, Danpanichkul P, Kulthamrongsri N, Kantagowit P (2025). Angiotensin-converting enzyme inhibitor-induced bowel angioedema: clinical features, diagnostic challenges, and recovery predictors from survival analysis: a systematic review of current reported cases. Ann Gastroenterol.

[10] Marmery H, Mirvis SE (2006). Angiotensin-converting enzyme inhibitor-induced visceral angioedema. Clin Radiol.

[11] Chandor S (2006). Tietz textbook of clinical chemistry and molecular diagnostics. JAMA.

[12] McPherson RA, Pincus MR (2021). Henry's Clinical Diagnosis and Management by Laboratory Methods. https://shop.elsevier.com/books/henrys-clinical-diagnosis-and-management-by-laboratory-methods/mcpherson/978-0-323-67320-4.

